# Study of andrographolide bioactivity against *Pseudomonas aeruginosa* based on computational methodology and biochemical analysis

**DOI:** 10.3389/fchem.2024.1388545

**Published:** 2024-04-12

**Authors:** Lihui He, Lai Song, Xuanhao Li, Shibo Lin, Guodong Ye, Huanxiang Liu, Xiaotian Zhao

**Affiliations:** ^1^ Department of Pharmacy, Chengdu Second Peoples Hospital, Chengdu, China; ^2^ Department of Oncology, Chengdu Second Peoples Hospital, Chengdu, China; ^3^ The Fifth Affiliated Hospital, Guangdong Provincial Key Laboratory of Molecular Target and Clinical Pharmacology, The NMPA and State Key Laboratory of Respiratory Disease, The School of Pharmaceutical Sciences, Guangzhou Medical University, Guangzhou, China; ^4^ Centre for Artificial Intelligence Driven Drug Discovery, Faculty of Applied Sciences, Macao Polytechnic University, Guangzhou, China

**Keywords:** andrographolide, biofilm, inhibitor, density functional theory, molecular docking, molecular dynamics

## Abstract

Andrographolide is one of the main biologically active molecules isolated from *Andrographis paniculata (A. paniculata)*, which is a traditional Chinese herb used extensively throughout Eastern Asia, India, and China. *Pseudomonas aeruginosa,* often known as *P. aeruginosa,* is a common clinical opportunistic pathogen with remarkable adaptability to harsh settings and resistance to antibiotics. *P. aeruginosa* possesses a wide array of virulence traits, one of which is biofilm formation, which contributes to its pathogenicity. One of the main modulators of the *P. aeruginosa*-controlled intramembrane proteolysis pathway is AlgW, a membrane-bound periplasmic serine protease. In this work, we have used a set of density functional theory (DFT) calculations to understand the variety of chemical parameters in detail between andrographolide and levofloxacin, which show strong bactericidal activity against *P. aeruginosa*. Additionally, the stability and interaction of andrographolide and levofloxacin with the protein AlgW have been investigated by molecular docking and molecular dynamics (MD) simulations . Moreover, the growth and inhibition of biofilm production by *P. aeruginosa* experiments were also investigated, providing insight that andrographolide could be a potential natural product to inhibit *P. aeruginosa.*

## 1 Introduction

*Andrographis paniculata (A. paniculata)*, named Chuanxinlian and belonging to the family of Acanthaceae, is a well-known traditional herb used in China and some other East Asian countries for a very long history and is highly valued for its therapeutic properties. Recently, research studies have revealed that *A. paniculata* has multifactorial pharmacological effects like anti-bacterial (Singha et al., 2003), anti-fungal (Sule et al., 2012), anti-viral (Wiart et al., 2005), anti-diabetic, and anti-cancer activities (Jayakumar et al., 2013). The diverse range of therapeutic properties of *A. paniculata* makes it a valuable herb in supporting immune health and fighting against various infections and diseases.

*A. paniculata* has a wide range of chemical components, including lactones, diterpenoids, diterpene glycosides, flavonoids, and flavonoid glycosides. Additionally, in previous work, we found that andrographolide, 14-deoxy-11,12-didehydroandrographolide, and neoandrographolide are the bioactive chemicals found in *A. paniculata* (Valdiani et al., 2017). Modern pharmacological research corroborates that the most effective major phytochemical constituent is andrographolide, which is the fundamental material basis for *A. paniculata*. Andrographolide is isolated from the stem and leaves of *A. paniculata*. Andrographolide possesses a unique molecular structure characterized by a bicyclic diterpene skeleton, which is the bioactive part of andrographolide (Gan et al., 2019).

Andrographolide has been demonstrated to be generally effective in treating allergic reactions, hemorrhagic lesions, and central nervous system dysfunction; in addition, it exhibits properties such as being an antipyretic, anti-inflammatory, hepatoprotective, and immunostimulant. Due to its various diverse pharmacological effects, the scientific community has paid close attention to andrographolide. Extensive research has identified andrographolide as a potent anti-inflammatory, antioxidant, and anti-microbial compound. The broad-spectrum anti-microbial activity of andrographolide is its most significant and initial therapeutic effect. Both Gram-positive and Gram-negative bacteria are present, and andrographolide has the ability to resist the growth of varieties of pathogens. Moreover, some studies have revealed that andrographolide has shown anti-viral activity against a range of viruses, including influenza, hepatitis B virus, and herpes (Yu et al., 2014; Gupta et al., 2017; Adiguna et al., 2021). In addition to the above properties, andrographolide acts as a potent and powerful anti-inflammatory agent. It inhibits the production of inflammatory mediators to reduce inflammation. In decade-long studies, andrographolide has also been displayed as a promising anti-cancer agent (Liang et al., 2008; Zhou et al., 2008). It has been discovered to impede the growth and multiplication of cancer cells and induce programmed cell death. In addition, andrographolide has been demonstrated to have the ability to modulate the immune system and enhance immune responses. In conclusion, andrographolide is a bioactive compound found in *A. paniculata* that offers a variety of medicinal properties. Its anti-inflammatory, antioxidant, antimicrobial, and anticancer properties make it a valuable natural compound with significant therapeutic potential, positioning it as a promising Chinese medicine worthy of further research and development.

According to earlier studies, andrographolide has been shown to inhibit the virulence and pathogenicity of *P. aeruginosa* (Banerjee et al., 2017a)*. P. aeruginosa,* a Gram-negative bacterial pathogen, caused a wide range of infections in humans, particularly in individuals with immunocompromised hosts. Because of its resistance to a variety of antibiotics, *P. aeruginosa* poses a challenge as a pathogen to treat. Up to now, there are some determined mechanisms regarding the resistance of *P. aeruginosa*: efflux pumps, inactivated enzymes, declined membrane permeability, altered target sites, and biofilm formation (Azam and Khan, 2019; Pang et al., 2019). The formation of biofilm is a significant factor that affects the mechanism of *P. aeruginosa* resistance. Biofilm, a complex and structured community of bacteria, adheres to surfaces and becomes embedded within a self-produced extracellular matrix. In addition, biofilm can protect *P. aeruginosa* from being distinguished by the innate immunity of the host and weaken the cleaning immune defense against *P. aeruginosa* pathogens. Meanwhile, a biofilm barrier could wrap the bacteria that block the effects of drugs, enhancing the resistance and tolerance of *P. aeruginosa* to anti-bacterial agents and phagocytes in the host. *P. aeruginosa* biofilms are composed of bacterial cells encased in the matrix, which consists of a combination of polysaccharides, proteins, DNA, and lipids (Flemming and Wingender, 2010). Alginate, one of three extracellular polysaccharides, is secreted to form biofilm in *P. aeruginosa*, and AlgU is the activator to produce alginate. The activated AlgU could increase the production of periplasmic chaperone proteins and other stress response proteins. MucA, the negative regulator of AlgU, is a key factor for *P. aeruginosa* mucus-type transformation (Brown et al., 2000; Bazire et al., 2010; Damron and Goldberg, 2012). This signaling pathway is mainly completed by the stepwise degradation of MucA through regulated intramembrane proteolysis (RIP) pathways. In the RIP pathway, the extracellular signal cascade proteolysis requires at least three different proteases, while the intercellular serine protease AlgW antagonizes the degradation of the periplasmic ends of σ factor MucA to initiate the RIP cascade reaction (Cezairliyan and Sauer, 2009). In addition, AlgW is suspected to influence the formation of biofilm in *P. aeruginosa.*

Notably, the goal of this study is to explore whether andrographolide directly inhibits *P. aeruginosa* growth or affects the formation of biofilm. As we know, levofloxacin is good at inhibiting *P. aeruginosa* growth, and it was chosen as our study control group. In order to clarify our hypothesis, we investigated andrographolide and levofloxacin at the molecular level by quantum chemical computations (Raghavachari, 2000; Spoel et al., 2005; Kryachko and Ludeña, 2014; van der), and the protein AlgW was chosen to study molecular dynamics with our title compounds. Finally, we concluded that andrographolide might be a potential inhibitor of *P. aeruginosa* as a traditional herb, and it also provided a new horizon in the pharmacology of Chinese medicine from a molecular perspective through computation methods.

## 2 Methods and materials

### 2.1 Quantum chemistry and electronic wavefunction analysis measurement

The molecular structure-optimized coordinates were performed utilizing Gaussian 16 software (Frisch et al., 2016.). The study utilized the def2TZVP basis set and M06-2X functional, both known for their high-quality theoretical approaches, and used solvent model density (SMD) for solvent modeling (Zhao and Truhlar, 2008; Hill, 2013). The software programs Gaussian View 6 and Origin 8 were utilized to construct the graphical representations. The frontier molecular orbitals (FMOs) (Bulat et al., 2004) were plotted in order to estimate the band gap energy. Additionally, the wavefunction input files for the title compounds were obtained via Multiwfn 3.8 wavefunction analyzer software (Lu and Chen, 2012a). The analyses of electrostatic potential (Murray and Politzer, 2011) and Fukui functions (Fukui, 1982) were also completed using Multiwfn 3.8. The VMD 1.9.1 application was utilized to generate all isosurface maps (Humphrey, et al., 1996) and Multiwfn output files.

### 2.2 Molecular docking

Molecular docking was used for the predictive combination mode of levofloxacin and andrographolide with the protein AlgW. According to Li et al. (2021), the structure of AlgW is from the Protein Data Bank (https://www.rcsb.org/), and its PDB code is 7CO2. Afterward, levofloxacin and andrographolide were downloaded from the PubChem database (https://pubchem.ncbi.nlm.nih.gov/) in. sdf format, and CIDs of levofloxacin and andrographolide are 149096 and 5318517, respectively. Levofloxacin was taken as a reference antibiotic in this study. All molecular docking operations were completed using Schrödinger 2022 software. First, the low-energy 3D structure of ligands was generated using the LigPrep panel (Mahto et al., 2014). Next, the protein preparation wizard module was used to take AlgW from its original state to the proper state for calculations, such as assigning bond orders, adding hydrogens, and H-bond assignment (Rolta et al., 2021). The protein binding sites were found by the sitemap panel. Finally, the Glide Ligand Docking Panel was used to run docking jobs using prepared proteins (Ashfaq et al., 2023).

### 2.3 Molecular dynamics

Molecular dynamic (MD) simulation monitored the motions of macromolecules over time (Hildebrand et al., 2019). The protein–ligand complex was simulated in the CHARMM36 force field and implemented using GROMACS 2020.6 software (Abraham, et al., 2015).

In order to generate the topology of compounds and force field parameters, the CGenFF server was used. The protein was put into a cube box of water of the simple point charge (spc216) model, and the distance between the protein peripherals and the box edge was 1 nm. Na^+^ and Cl^−^ions were added to neutralize the charge on the complex and maintain its electrical neutrality. Then, the energy of the MD system was minimized by steepest descent minimization. The system temperature was stabled by the NVT (number of particles, volume, and temperature) ensemble, and the system pressure was stabled by the NPT (number of particles, pressure, and temperature) ensemble. Finally, position restraints were released, and production MD was run to collect data at 100 ns (Hou et al., 2023).

### 2.4 Bacterial growth from optical density measurement *in vitro*

The overnight PAO1 was diluted to a final optical density (OD_600_) of 0.05 in LB broth. The cell suspensions were taken every hour, and the cell density was determined by measuring the absorbance value at 600 nm using a microplate reader to monitor the growth of bacteria.

### 2.5 Biofilm formation assay

As previously mentioned, biofilm formation was quantified using crystal violet staining (Scoffield et al., 2017). In brief, overnight PAO1 was 100 times diluted in brand-new M63 minimal medium that has been enhanced with arginine and magnesium sulfate and then incubated for 12–16 h at 37 °C. A volume of 1 mL of diluted cell suspension was transferred into a 24-well plate, and different concentrations of andrographolide and levofloxacin were added, followed by incubation for 48 h. Only untreated cultures that contained 0.625% DMSO were used as a negative control. After incubation, wells were washed with sterilized phosphate-buffered saline (PBS) three times to remove the M63 minimal medium. The adhered cells were stained with 0.05% crystal violet for 30 min at room temperature, followed by washing the wells three times with PBS. Ethanol (95%) was used to elute the biofilm, and the absorbance of the eluted solution was determined at 570 nm using a microplate reader. The assay for crystal violet was measured three times. The difference between the three mean values was calculated using the one-sample *t*-test; *p* < 0.01 was considered statistically significant.

## 3 Results and discussion

### 3.1 FMO analysis and electronic parameters

In order to understand the molecular chemical reactivity, molecular stability, electronic characteristics, and optical properties of molecules, the frontier molecular orbitals [highest occupied molecular orbital (HOMO)–least unoccupied molecular orbital (LUMO)] (Bredas, 2014) are often employed. When the **Δ**E value is large, the HOMO–LUMO energy gap is less, which suggests that the chemical reactivity is lower (Gopalakrishnan et al., 2014). Band gap energies are computed, and the HOMO–LUMO is given an estimate using a def2TZVP functional based solvation methodology for several polar solvents (DMSO and water).

**FIGURE 1:**
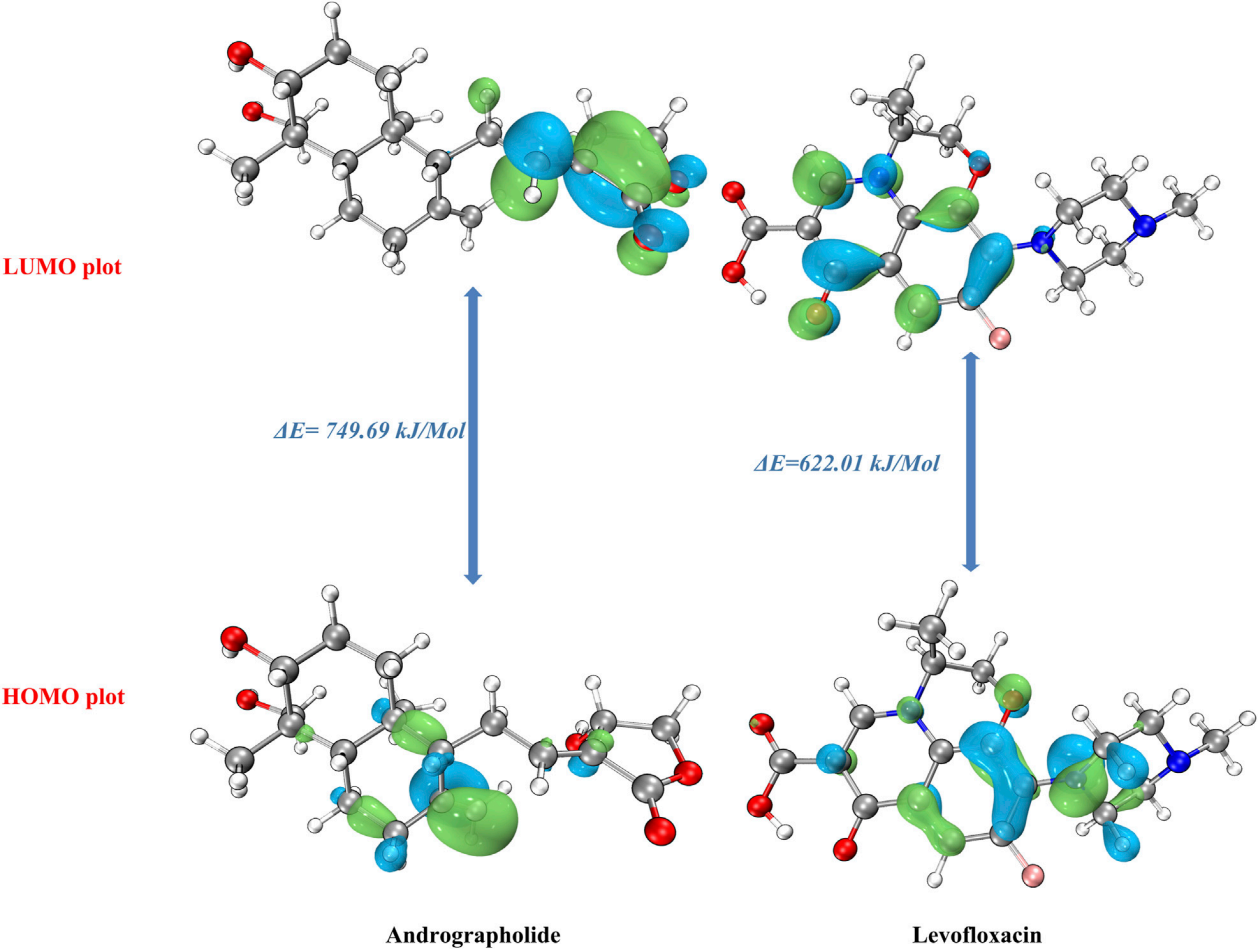
Frontier molecular orbital of the andrographolide and levofloxacin compounds.

In order to acquire a greater comprehension of the chemical properties of the title’s molecules, the electronic parameters were investigated. These data were obtained by conceptual DFT analysis from the Multiwfn 3.8 program. Based on the data presented in Table 1, it can be observed that both the HOMO and LUMO, together with their adjacent orbitals, possess negative energies, which suggests that the molecule is in a stable state (Zhou and Parr, 1990). Figure 1 illustrates the energy gap in the gas phase. The title’s compounds have energy gaps, with values of 749.69 and 622.01 kJ/mol, respectively. A positive reflection of its molecular electrical transport capabilities was observed in the levofloxacin compound’s ΔE at lower values. In addition, the electron cloud primarily associated with levofloxacin is located on the carbon–carbon bond of the ring in the LUMO that corresponds to π→π*. Additional information and details can be found in Supplementary Table S1.

**TABLE 1:** Frontier molecular orbital energy and reactivity characteristic parameters of title compounds.

Parameter	Andrographolide			Levofloxacin		
	Gas	Water	DMSO	Gas	Water	DMSO
HOMO (eV)	−8.253	−8.121	−8.075	−7.502	−7.264	−7.113
LUMO (eV)	−0.483	−0.508	−0.309	−1.055	−0.958	−0.805
Ionization potential (I)	8.455	6.363	6.356	7.567	5.526	5.496
Electron affinity (A)	0.147	2.286	1.899	0.748	2.593	2.246
Energy gap (**Δ**E gap eV)	7.770	7.613	7.766	6.447	6.306	6.308
Electronegativity (χ)	4.301	4.325	4.128	4.158	4.060	3.871
Chemical potential (μ)	−4.301	−4.325	−4.128	−4.158	−4.060	−3.871
Chemical hardness (η)	8.308	4.077	4.457	6.819	2.933	3.251
Chemical softness (ζ)	0.120	0.245	0.224	0.147	0.341	0.308
Electrophilicity index (ω)	1.114	2.293	1.911	1.267	2.809	2.305

$I=-E_{HOMO},$ $A=-E_{LUMO},$ $\eta =\frac{I-A}{2},$ $\chi =\frac{I+A}{2},$ $\mu =-\frac{I+A}{2},$ $\omega =\frac{\mu 2}{2\eta },$ and $\zeta =\frac{1}{2\eta }.$

There are many quantum chemical parameters in Koopmans’s theory (Luo et al., 2006). The following parameters are shown in Table 1: ionization energies (I), electron affinities (A), electronegativity (χ), chemical hardness (η), chemical softness (ζ), chemical potential (μ), and the global electrophilicity index (ω). According to Table 1, the chemical hardness (η) value (Parr and Pearson, 1983), the gas phase exhibits the largest degree of andrographolide, suggesting a greater level of chemical stability and reactivity. Despite this, when dissolved in water, we achieved the highest chemical softness measurement (2.809), indicating that levofloxacin exhibits greater reactivity in this specific solvent. Furthermore, the title compounds were evaluated for solvent effects, revealing that polar solvents (water and DMSO) could decrease the chemical hardness (η) value, which is much lower than in the gas phase.

Furthermore, when comparing the energy gap, we found that the levofloxacin compound had the lowest value at 6.306 eV in a water solvent. The molecules are harder when the HOMO–LUMO energy gap is larger (Chattaraj and Maiti, 2003). Conversely, it exhibits a negative correlation with chemical reactivity and softness. Theoretical parameter data have shown that the andrographolide is more reactive in polar solvents, but it may not exhibit higher biological activity than the levofloxacin compound.

### 3.2 Reactivity site analysis

#### 3.2.1 Electrostatic potential analysis

In order to comprehend the sites of electrophilic (negative) and nucleophilic (positive) chemical reactions and hydrogen bond interactions, it is crucial to conduct molecular electrostatic potential (MEP) surface analyses (Ayers et al., 2002; Pino-Rios et al., 2019). Furthermore, it provides information regarding the dimensions, configuration, active regions, and electrical charge distribution of the molecule. MEP surfaces for the chosen molecules were generated using the M0-62x/def2TZVP method.

**FIGURE 2:**
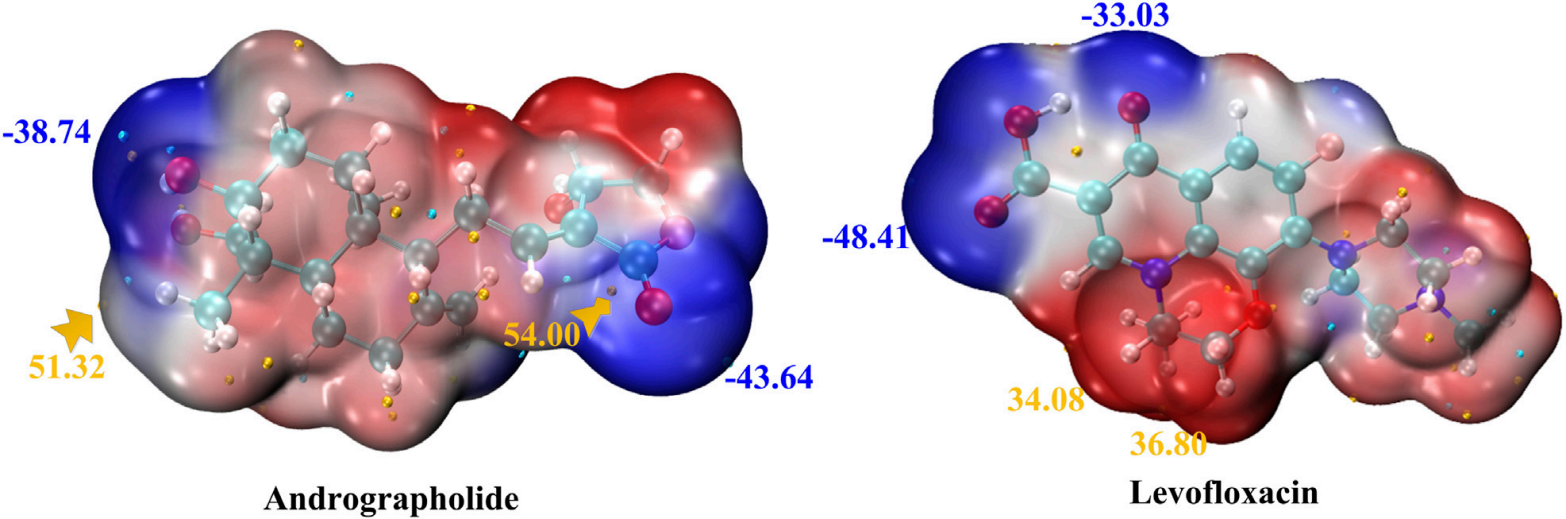
MEP of andrographolide and levofloxacin using the DFT method. The ESP’s notable surface low and high points are depicted as gold and cyan spheres (unit: kcal/mol).

MEP maps for title compounds in the gas phase are displayed in Figure 2. The cyan and yellow balls, which represented the negative and positive electrostatic potential (ESP) surface values, respectively, were meant to symbolize the various electrostatic potential surface values. The electrophilic site is shown in red on the surface diagram, whereas the nucleophilic site is displayed in blue. The andrographolide and levofloxacin molecules are shown in a blue color (representing the nucleophilic site) above the oxygen atom. The blue color also indicates the nucleophilic reactivity of carbon and hydrogen in the molecule being studied. It may be inferred that the biological activity of a molecule can be predicted based on its active oxygen site.

Figure 2 displays that the ESP-mapped molecular vdW surface of carboxyl in the levofloxacin monomer is more deeply blue. We acquired a more intense negative value for ESP of −48.41 kcal/mol. Given its superior ability to attract electrophiles, the site with a higher negative ESP is more likely to be reactive. In Figure 2, from the left zone in the andrographolide part, it is evident that the oxygen-containing surface exhibited the lowest ESP value, while the vdW surface exhibited a notably negative ESP value. The molecular surface area of andrographolide is at its lowest value of −43.64 kcal/mol. The results clearly demonstrated that the andrographolide compound exhibits electrophilic reactivity but not to a greater extent than levofloxacin.

#### 3.2.2 Fukui functions and dual descriptor

**FIGURE 3:**
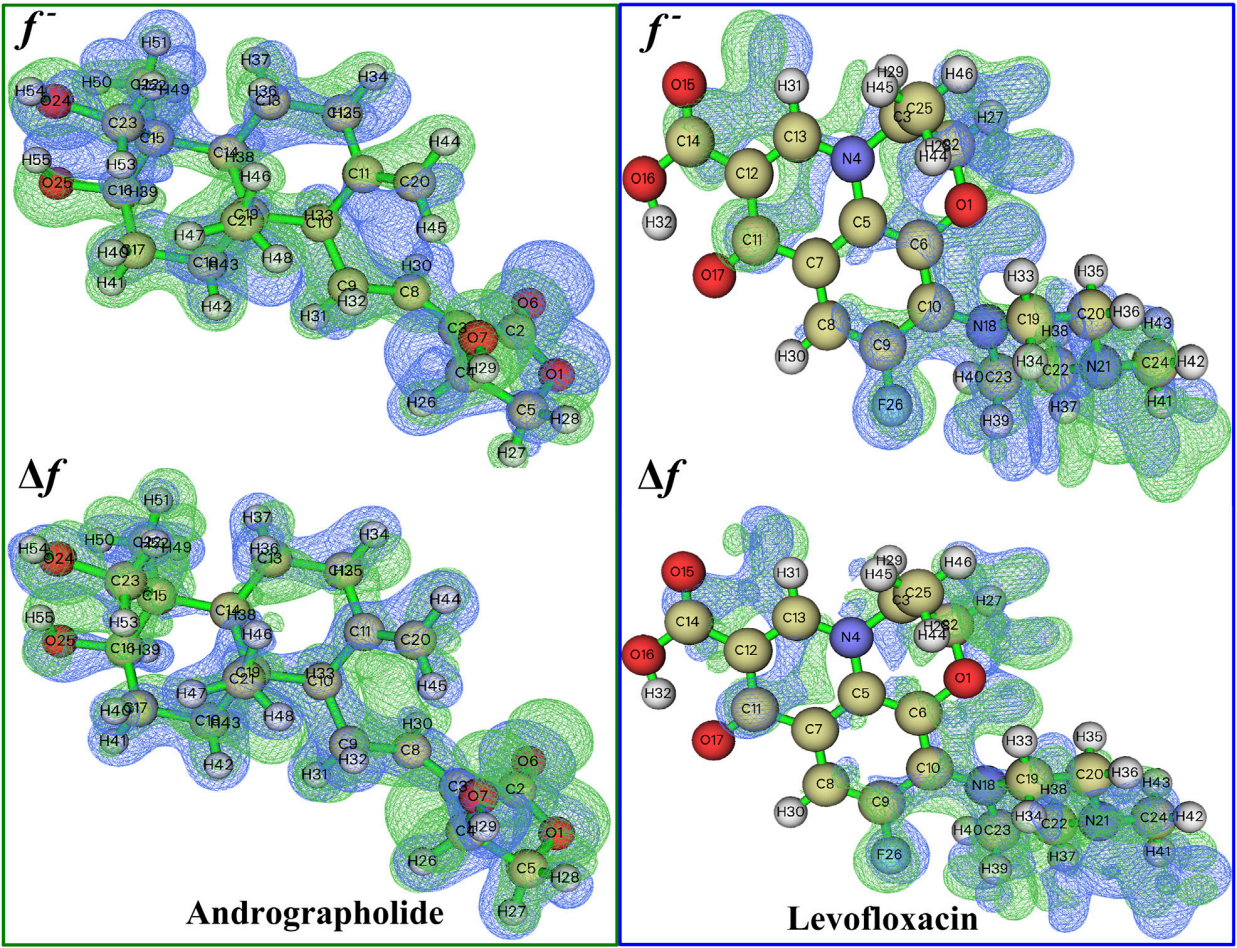
A visual representation of the relationship between Fukui functions and the dual descriptor of the title compound at 0.05 isovalue, respectively.

The chemical reactivity of andrographolide was analyzed using the Fukui function and dual descriptor (Lu and Chen, 2012b) to figure out electrophilic attack sites. An analysis was conducted on the local electrophilicity, dual descriptors, and Fukui functions of all atomic sites. The graphical representation of Fukui functions and dual descriptors can be observed in Figure 3, with an isovalue of 0.05, where blue regions represent negative regions that are susceptible to nucleophilic attack, while the green dots indicate positive regions that are vulnerable to electrophilic attack. The dual descriptor (Morell et al., 2005) (**Δ**f(r)) is shown as follows:

$$f\left(r\right)={\left[\frac{\partial \rho \left(r\right)}{\partial N}\right]}_{V},$$ (1)

$$f^{+}\left(r\right)=\rho_{N+1}\left(r\right)-\rho_{N}\left(r\right),$$ (2)

$$f^{-}\left(r\right)=\rho_{N}\left(r\right)-\rho_{N-1}\left(r\right),$$ (3)

$$f^{0}\left(r\right)=\frac{f^{+}\left(r\right)+f^{-}\left(r\right)}{2},$$ (4)

where N represents the total number of electrons in the current system and makes the external potential the constant component in the partial derivative. The dual descriptor is an additional effective approach for detecting reactive sites. The Fukui function and dual descriptor **Δ**f are closely interconnected from a formal perspective:

$$∆f\left(r\right)=f^{+}\left(r\right)-f^{-}\left(r\right).$$ (5)

Unlike the Fukui function, both types of reactive sites may be exposed simultaneously via **f**. If **Δ**f > 0, the site is considered beneficial for an electrophilic attack; otherwise, it is considered acceptable for a nucleophilic attack.

The more positive atomic site’s f^-^ value, as indicated by Supplementary Table S2, is C20 in 0.1954. In addition, the majority of the f^-^ function’s positive area is concentrated on C11, C20, and H44, indicating that these are attractive reactive sites for electrophilic attack. Compared to levofloxacin, it is easily found that f^−^ function is placed on C_6_, C_7_, and N_18_, especially on N_18,_ whose value reached 0.1323.

The dual descriptor function Δf value, as shown in Supplementary Table S2, indicates that the electrophilic nature of the atomic site is C20 in −0.1930 (less than 0), and H29 is the nucleophilic attack type at the atomic location in andrographolide. In the levofloxacin part of Supplementary Table S3, the electrophilic properties of the atomic site are N18 in −0.1145 and the nucleophilic attack type at the atomic location is C11 in 0.0732. It is evident that the surrounding hydrogen atoms’ nucleophilic characteristics are caused by the oxygen and nitrogen atoms. Figure 3 definitely illustrates the remaining part of the local electrophilicity of the atomic location.

### 3.3 Molecular docking and molecular dynamics

**FIGURE 4:**
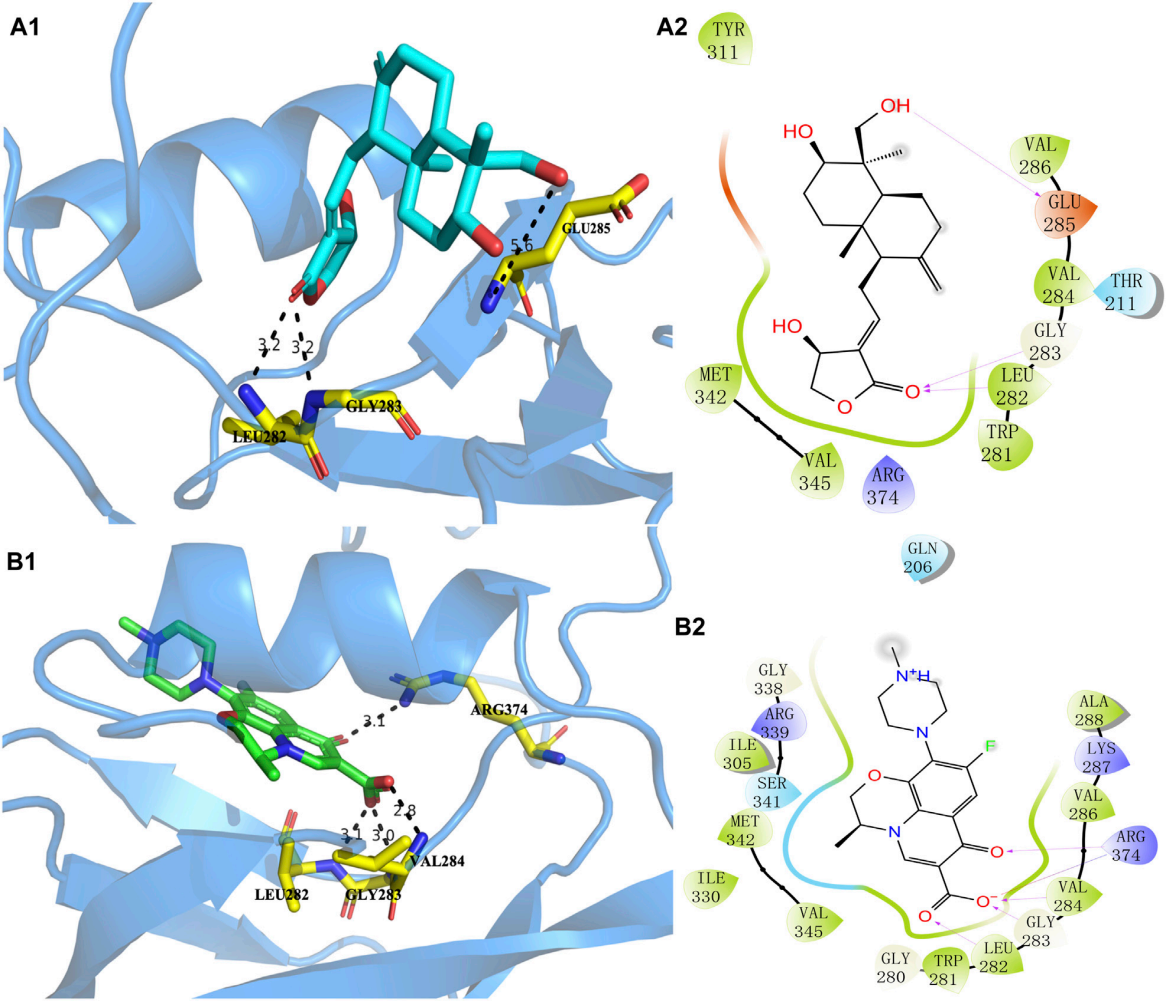
Molecular interaction of andrographolide and levofloxacin with AlgW, respectively. Label **(A1)** represents the 3D structure of complex andrographolide–AlgW, and **(A2)** represents their 2D structure. Labels **(B1, B2)** are related to the levofloxacin–AlgW complex.

The higher the absolute value of the Glide GScore, the stronger the potential affinity between the molecule and protein. The Glide GScores of levofloxacin and andrographolide with the protein AlgW were −8.030 and −3.261, respectively. The Glide GScore showed that the affinity between levofloxacin and protein binding is greater than that of andrographolide. Figure 4; Supplementary Table S4 show andrographolide (Labels a1 and a2) and levofloxacin (Labels b1 and b2) binding conformations of AlgW. It showed that around the active pocket, small molecules mainly interact with amino acid residues of proteins at the distance of 4 Å through hydrogen bonding and van der Waals forces.

The 3D docking diagram of andrographolide and levofloxacin with AlgW showed that AlgW could be attached to the active pocket of the protein. Although the absolute value of the Glide GScore between andrographolide and AlgW is smaller than that between levofloxacin and AlgW, andrographolide is a potential inhibitor of AlgW due to the Glide GScore of −3.261.

**FIGURE 5:**
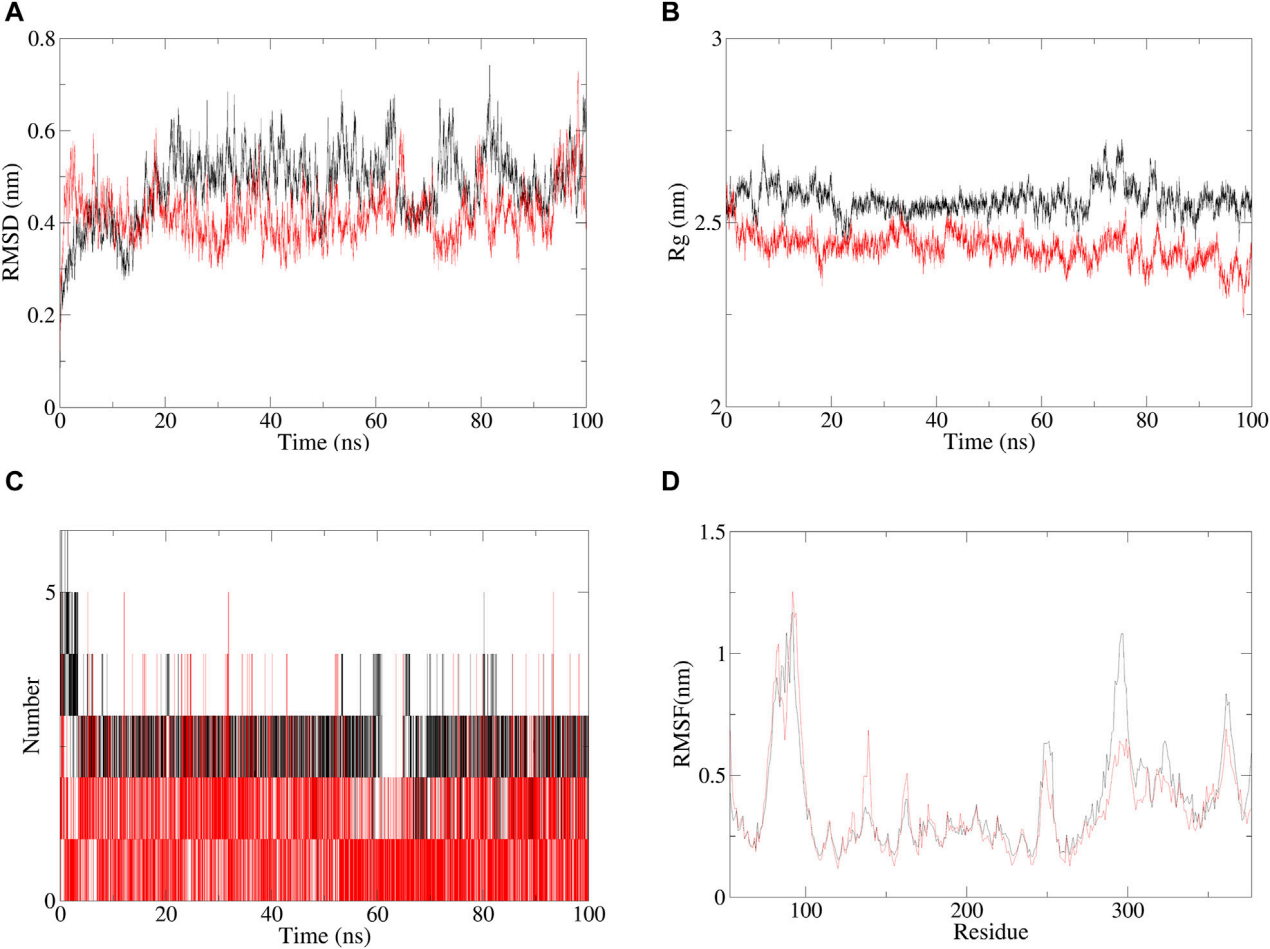
Specific information of RMSD **(A)**, gyration radius**(B)**, hydrogen bonding of complexes **(C)**, and RMSF plot **(D)**, red line represents andrographolide–AlgW, and black line represents levofloxacin–AlgW.

The molecular dynamics evaluation parameters for andrographolide–AlgW (red line) and levofloxacin–AlgW (black line) complexes are shown in Figure 5. RMSD can quantify the stability of protein–ligand complex structures over a certain period of time (Rudnev et al., 2022). In this study, the RMSD of andrographolide–AlgW and levofloxacin–AlgW were calculated within 100 ns. The RMSD analysis showed that the RMSD value of levofloxacin-AlgW was balanced after 20 ns. However, the RMSD value of andrographolide-AlgW began to stabilize after 10 ns. It was in equilibrium between 10 ns and 90 ns. After 90 ns, there was a slight increase in the RMSD value. The gyration radius reflected the volume and shape of the complex. The larger the turning radius of the same system, the more expansive the system becomes (Yanao et al., 2007). It showed that levofloxacin–AlgW was more expansive than andrographolide–AlgW. This study analyzed the variation in the number of hydrogen bonds between ligands and proteins over time. In molecular dynamics, the hydrogen bond number of andrographolide–AlgW was not better than that of levofloxacin–AlgW. RMSF was used to calculate the root mean square fluctuations of atomic positions in trajectories (Thangavel and Albratty, 2023). The results indicated that there was little fluctuation in the atomic positions of amino acids around the docking site of the AlgW protein. The molecular dynamics results indicated that in a 100 ns simulation, the binding of andrographolide AlgW and levofloxacin AlgW complexes was stable and persistent.

### 3.4 Growth of *P. aeruginosa* in the presence of different concentrations of andrographolide

**FIGURE 6:**
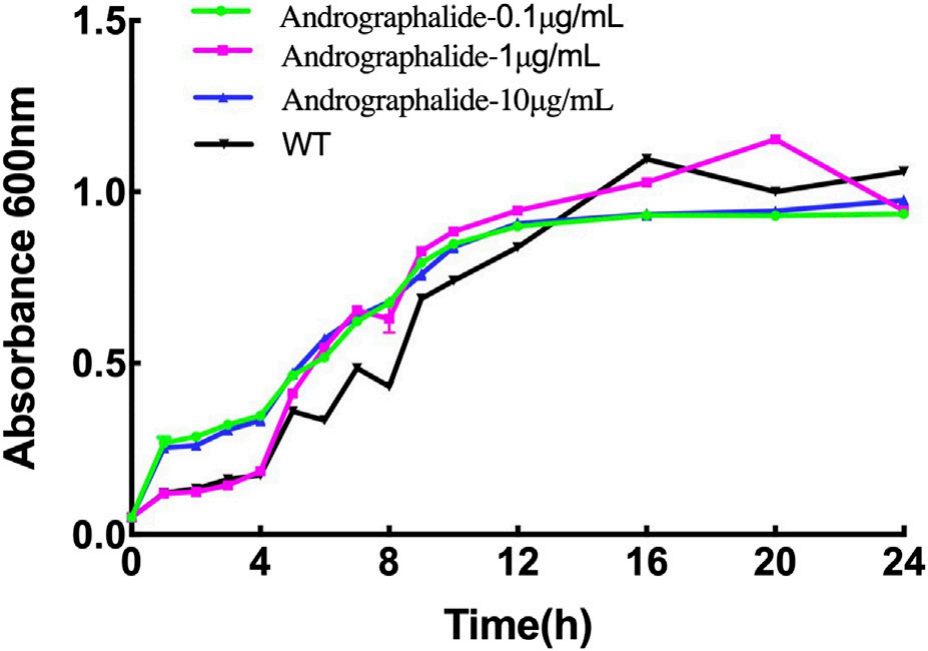
24-h growth curves of *P. aeruginosa* with different treatments: (1) bacteria only, (2) 0.1 μg/mL andrographolide, (3)10 μg/mL andrographolide, and (4) 100 μg/mL andrographolide. The OD values of the cultivated bacteria were measured at λ = 600 nm in the LB medium.

In order to explore the effect of *P. aeruginosa* growth with different concentrations of andrographolide, the bacteria were grown in Luria–Bertani broth (LB) agar medium for 24 h, and the results are summed up in Figure 6. The results showed that there is no inhibitory effect on bacterial growth compared with the wild type with 0.1 μg/mL, 10 μg/mL, or 100 μg/mL of andrographolide. In addition, it is reasonable to explain that andrographolide does not directly sterilize the bacterial like antibiotics, and it gave us some clues that andrographolide may have effects on components like biofilm, virulence, quorum sensing (QS) systems, or secretion systems.

### 3.5 Inhibition of biofilm production of *P. aeruginosa* by andrographolide

**FIGURE 7:**
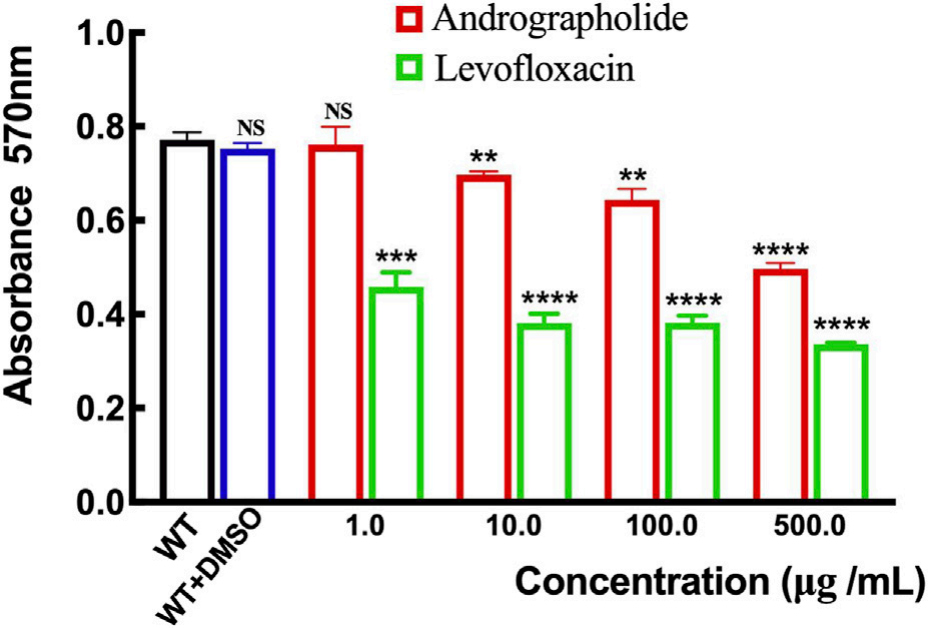
Comparison of the effect of andrographolide and levofloxacin on biofilm formation with different concentrations of 1.0, 10.0, 100.0, and 500.0 μg/mL utilizing assays on 24-well crystal violet microtiter plate assays at λ = 570 nm. Standard deviations are represented by error bars. Three samples were considered statistically meaningful, with a *p*-value of less than 0.01.

Although there is no evidence to prove the activity of andrographolide against *P. aeruginosa* growth assay, previous studies have shown that andrographolide and 14-deoxy-11,12-didehydroandrographolide may influence the formation of biofilm in *P. a*eruginosa (Majumdar et al., 2020). To determine whether andrographolide will inhibit the biofilm of *P. aeruginosa*, we compare the wild-type *P. aeruginosa* (PAO1) with different concentrations of andrographolide and positive control levofloxacin, which can inhibit the formation of biofilm, by staining adherent cells with crystal violet to investigate biofilm formation (Banerjee et al., 2017a). Figure 7 shows that andrographolide with different concentrations has some effect on biofilm formation, although it is less effective than levofloxacin at the same concentration.

## 4 Conclusion

The traditional Chinese herb *A. paniculata* and one of its bioactive compounds, andrographolide, have been studied for their anti-cancer, anti-bacterial, anti-fungal, and anti-viral functions. In addition, *P. aeruginosa*, a common opportunistic pathogen, has several resistance mechanisms, one of which is biofilm formation. Biofilms are the protective structures that can make the bacterium more resistant to antibiotics and immune responses. In this study, we used computational methods based on the structure of chemistry at the molecular level, suggesting that andrographolide may be a potential inhibitor. We have compared andrographolide with levofloxacin, which is effective against *P. aeruginosa*, and reported that andrographolide has a certain effect on the formation of the *P. aeruginosa* biofilm, although not as effectively as levofloxacin. Furthermore, we can speculate whether other bioactive ingredients of *A. paniculata* have similar effects to andrographolide, and we could explore the specific mechanism of andrographolide affecting biofilm formation at the genetic level. In conclusion, andrographolide has been proved to influence biofilm formation, but it is necessary to note that additional investigation and rigorous clinical studies are necessary to comprehensively evaluate their effectiveness, safety, and potential applications in the treatment of *P. aeruginosa* infections.

## Data Availability

The original contributions presented in the study are included in the article/Supplementary Material; further inquiries can be directed to the corresponding author.
