# Supplementary material for: Study of andrographolide bioactivity against Pseudomonas aeruginosa based on computational methodology and biochemical analysis
Source: Front Chem. 2024 Apr 12;12:1388545. doi: 10.3389/fchem.2024.1388545 (PMC11045890; doi:10.3389/fchem.2024.1388545)
Supplement: Supplementary file 1 [file DataSheet1.docx]

# Study of Andrographolide bioactivity with *Pseudomonas aeruginosa* by Computational Methodology and Biochemical Analysis

Lihui He ^a†^, Lai Song ^b†^, Xuanhao Li ^a^, Shibo Lin ^a^, Guodong Ye ^c^, Huanxiang Liu ^d^, Xiaotian Zhao ^a^*****

^a^ *Department of Pharmacy, Chengdu Second Peoples Hospital, Chengdu 610017, China.*

^b^ *Department of Oncology, Chengdu Second Peoples Hospital, Chengdu 610017, China.*

^c^ *The Fifth Affiliated Hospital, Guangdong Provincial Key Laboratory of Molecular Target & Clinical Pharmacology, the NMPA and State Key Laboratory of Respiratory Disease, The School of Pharmaceutical Sciences, Guangzhou Medical University, Guangzhou, 511436, China.*

^d^*Centre for Artificial Intelligence Driven Drug Discovery, Faculty of Applied Sciences, Macao Polytechnic University，Macao 999078, China.*

† *These authors have contributed equally to this work*

***Correspondence**:

Xiaotian Zhao
Tel: +86-15769207006
E-mail: [cshpharmcyzxt@foxmail.com](mailto:cshpharmcyzxt@foxmail.com)

## Quantum Chemical Parameters

The chemical reactivity descriptors were calculated using DFT. These are very important physical parameters to understand chemical and biological activities of the phytochemical constituents. The calculated HOMO-LUMO orbital energies can be used to estimate the ionization energy [1], electron affinity [1], electronegativity [2], electronic chemical potential [2], molecular hardness [3], molecular softness [3], and electrophilicity index [4] using the following equations: ionization energy (IE) = −𝜀HOMO, electron affinity (EA) = −𝜀LUMO, electronegativity (𝜒) = (IE + EA)/2, electronic chemical potential (𝜇) = −𝜒, chemical hardness (𝜂) = (IE− EA)/2, chemical softness (𝜎) = 1/𝜂, electrophilicity index (𝜔) = 𝜇2 /2

References:

[1] J. B. Foresman and A. Frisch, Exploring Chemistry with Electronic Structure Methods, Gaussian, Pittsburg, Pa, USA, 1995.

[2] L. Pauling, The Nature of the Chemical Bond, Cornell University Press, Ithaca, NY, USA, 1960.

[3] P. Senet, “Chemical hardnesses of atoms and molecules from frontier orbitals,” Chemical Physics Letters, vol. 275, no. 5-6, pp. 527–532, 1997.

[4] R. G. Parr and R. G. Pearson, “Absolute hardness: companion parameter to absolute electronegativity,” Journal of the American Chemical Society, vol. 105, no. 26, pp. 7512–7516, 1983.

## Tables

**Table S1** The calculated quantum chemical Hirshfeld charges and Fukui Function of title compounds.

|  | **Andrograplide** | **Levofloxacin** |
| --- | --- | --- |
| **HOMO** | -8.253 eV | -7.502 eV |
| **LUMO** | -0.483 eV | -1.055 eV |
| **HOMO-1** | -8.749 eV | -7.770 eV |
| **LUMO+1** | 1.001 eV | -0.603 eV |
| **△*E*_HOMO-LUMO_** | 7.770 eV | 6.447 ev |
| **△*E*_HOMO-LUMO_** | 749.686 kJ/mol | 622.014 kJ/mol |

**Table S2** The calculated quantum chemical Hirshfeld charges and Fukui Function of Andrographolide.

| Atom Number | q_(N)_ | q_(N+1)_ | q_(N-1)_ | f^-^ | f^+^ | f^0^ | **Δ**f |
| --- | --- | --- | --- | --- | --- | --- | --- |
| 1(O ) | -0.1341 | -0.2008 | -0.1142 | 0.0199 | 0.0667 | 0.0433 | 0.0467 |
| 2(C ) | 0.1953 | 0.1101 | 0.2007 | 0.0053 | 0.0852 | 0.0453 | 0.0799 |
| 3(C ) | -0.0375 | -0.1205 | -0.0174 | 0.0201 | 0.0830 | 0.0515 | 0.0630 |
| 4(C ) | 0.0468 | 0.0242 | 0.0524 | 0.0056 | 0.0226 | 0.0141 | 0.0171 |
| 5(C ) | 0.0262 | -0.0024 | 0.0376 | 0.0115 | 0.0285 | 0.0200 | 0.0171 |
| 6(O ) | -0.2771 | -0.4011 | -0.2548 | 0.0223 | 0.1239 | 0.0731 | 0.1016 |
| 7(O ) | -0.2105 | -0.2443 | -0.2023 | 0.0083 | 0.0338 | 0.0210 | 0.0255 |
| 8(C ) | 0.0157 | -0.1186 | -0.0000 | -0.0157 | 0.1343 | 0.0593 | 0.1500 |
| 9(C ) | -0.0454 | -0.0667 | -0.0402 | 0.0052 | 0.0213 | 0.0132 | 0.0161 |
| 10(C ) | -0.0135 | -0.0156 | -0.0039 | 0.0096 | 0.0021 | 0.0058 | -0.0075 |
| 11(C ) | 0.0018 | -0.0025 | 0.1361 | 0.1343 | 0.0043 | 0.0693 | -0.1300 |
| 12(C ) | -0.0498 | -0.0546 | -0.0312 | 0.0186 | 0.0049 | 0.0117 | -0.0137 |
| 13(C ) | -0.0511 | -0.0560 | -0.0280 | 0.0232 | 0.0049 | 0.0140 | -0.0183 |
| 14(C ) | -0.0137 | -0.0149 | -0.0138 | -0.0001 | 0.0012 | 0.0005 | 0.0013 |
| 15(C ) | 0.0070 | 0.0065 | 0.0110 | 0.0040 | 0.0005 | 0.0023 | -0.0036 |
| 16(C ) | 0.0493 | 0.0476 | 0.0526 | 0.0033 | 0.0016 | 0.0025 | -0.0017 |
| 17(C ) | -0.0612 | -0.0645 | -0.0529 | 0.0083 | 0.0033 | 0.0058 | -0.0051 |
| 18(C ) | -0.0513 | -0.0517 | -0.0426 | 0.0087 | 0.0004 | 0.0045 | -0.0082 |
| 19(C ) | 0.0188 | 0.0175 | 0.0407 | 0.0219 | 0.0013 | 0.0116 | -0.0206 |
| 20(C ) | -0.0943 | -0.0968 | 0.1011 | 0.1954 | 0.0024 | 0.0989 | -0.1930 |
| 21(C ) | -0.0917 | -0.0954 | -0.0860 | 0.0057 | 0.0037 | 0.0047 | -0.0020 |
| 22(C ) | -0.0919 | -0.0954 | -0.0861 | 0.0058 | 0.0035 | 0.0046 | -0.0023 |
| 23(C ) | 0.0229 | 0.0222 | 0.0243 | 0.0014 | 0.0008 | 0.0011 | -0.0006 |
| 24(O ) | -0.1932 | -0.2014 | -0.1803 | 0.0129 | 0.0082 | 0.0105 | -0.0047 |
| 25(O ) | -0.2460 | -0.2564 | -0.2278 | 0.0182 | 0.0104 | 0.0143 | -0.0078 |
| 26(H ) | 0.0348 | 0.0028 | 0.0463 | 0.0116 | 0.0320 | 0.0218 | 0.0204 |
| 27(H ) | 0.0416 | 0.0100 | 0.0566 | 0.0149 | 0.0316 | 0.0233 | 0.0167 |
| 28(H ) | 0.0450 | 0.0130 | 0.0566 | 0.0116 | 0.0321 | 0.0218 | 0.0205 |
| 29(H ) | 0.1615 | 0.1211 | 0.1761 | 0.0147 | 0.0404 | 0.0275 | 0.0257 |
| 30(H ) | 0.0515 | -0.0023 | 0.0556 | 0.0040 | 0.0539 | 0.0290 | 0.0498 |
| 31(H ) | 0.0398 | 0.0057 | 0.0583 | 0.0185 | 0.0341 | 0.0263 | 0.0156 |
| 32(H ) | 0.0324 | 0.0129 | 0.0390 | 0.0066 | 0.0195 | 0.0130 | 0.0129 |
| 33(H ) | 0.0283 | 0.0223 | 0.0563 | 0.0280 | 0.0060 | 0.0170 | -0.0221 |
| 34(H ) | 0.0350 | 0.0247 | 0.0607 | 0.0257 | 0.0103 | 0.0180 | -0.0155 |
| 35(H ) | 0.0319 | 0.0258 | 0.0683 | 0.0364 | 0.0061 | 0.0213 | -0.0303 |
| 36(H ) | 0.0246 | 0.0192 | 0.0373 | 0.0127 | 0.0053 | 0.0090 | -0.0074 |
| 37(H ) | 0.0311 | 0.0212 | 0.0557 | 0.0246 | 0.0100 | 0.0173 | -0.0147 |
| 38(H ) | 0.0242 | 0.0229 | 0.0334 | 0.0093 | 0.0013 | 0.0053 | -0.0080 |
| 39(H ) | 0.0263 | 0.0249 | 0.0316 | 0.0053 | 0.0014 | 0.0033 | -0.0039 |
| 40(H ) | 0.0232 | 0.0180 | 0.0316 | 0.0084 | 0.0052 | 0.0068 | -0.0032 |
| 41(H ) | 0.0341 | 0.0271 | 0.0506 | 0.0165 | 0.0070 | 0.0118 | -0.0095 |
| 42(H ) | 0.0278 | 0.0281 | 0.0394 | 0.0116 | -0.0003 | 0.0056 | -0.0119 |
| 43(H ) | 0.0269 | 0.0275 | 0.0375 | 0.0106 | -0.0006 | 0.0050 | -0.0112 |
| 44(H ) | 0.0342 | 0.0191 | 0.0918 | 0.0576 | 0.0152 | 0.0364 | -0.0424 |
| 45(H ) | 0.0246 | 0.0291 | 0.0616 | 0.0371 | -0.0046 | 0.0162 | -0.0416 |
| 46(H ) | 0.0264 | 0.0202 | 0.0310 | 0.0046 | 0.0062 | 0.0054 | 0.0015 |
| 47(H ) | 0.0252 | 0.0150 | 0.0450 | 0.0198 | 0.0102 | 0.0150 | -0.0095 |
| 48(H ) | 0.0279 | 0.0311 | 0.0345 | 0.0066 | -0.0032 | 0.0017 | -0.0097 |
| 49(H ) | 0.0290 | 0.0269 | 0.0343 | 0.0053 | 0.0021 | 0.0037 | -0.0032 |
| 50(H ) | 0.0299 | 0.0228 | 0.0428 | 0.0130 | 0.0071 | 0.0100 | -0.0059 |
| 51(H ) | 0.0270 | 0.0225 | 0.0333 | 0.0063 | 0.0045 | 0.0054 | -0.0017 |
| 52(H ) | 0.0274 | 0.0257 | 0.0287 | 0.0013 | 0.0017 | 0.0015 | 0.0004 |
| 53(H ) | 0.0232 | 0.0234 | 0.0247 | 0.0014 | -0.0002 | 0.0006 | -0.0016 |
| 54(H ) | 0.1668 | 0.1593 | 0.1793 | 0.0125 | 0.0075 | 0.0100 | -0.0049 |
| 55(H ) | 0.1173 | 0.1115 | 0.1269 | 0.0096 | 0.0058 | 0.0077 | -0.0038 |

Nucleophilic attack **:** f^+^= q_(N)_- q_(N+1)_ Electrophilic attack : f^-^= q_(N-1)_-q_(N)_  Radical attack: f^0^= (q_(N-1)_- q_(N+1)_)/2

**Table S3** The calculated quantum chemical Hirshfeld charges and Fukui Function of levofloxacin.

| Atom Number | q_(N)_ | q_(N+1)_ | q_(N-1)_ | f^-^ | f^+^ | f^0^ | **Δ**f |
| --- | --- | --- | --- | --- | --- | --- | --- |
| 1(O ) | -0.1188 | -0.1447 | -0.0804 | 0.0384 | 0.0259 | 0.0322 | -0.0125 |
| 2(C ) | 0.0349 | 0.0216 | 0.0520 | 0.0172 | 0.0133 | 0.0152 | -0.0039 |
| 3(C ) | 0.0359 | 0.0255 | 0.0424 | 0.0065 | 0.0104 | 0.0084 | 0.0039 |
| 4(N ) | -0.0002 | -0.0334 | 0.0021 | 0.0022 | 0.0333 | 0.0177 | 0.0311 |
| 5(C ) | 0.0269 | 0.0095 | 0.0356 | 0.0087 | 0.0174 | 0.0130 | 0.0087 |
| 6(C ) | 0.0506 | 0.0165 | 0.1090 | 0.0584 | 0.0342 | 0.0463 | -0.0242 |
| 7(C ) | -0.0302 | -0.0415 | 0.0106 | 0.0407 | 0.0113 | 0.0260 | -0.0294 |
| 8(C ) | -0.0489 | -0.1051 | -0.0205 | 0.0284 | 0.0562 | 0.0423 | 0.0278 |
| 9(C ) | 0.0850 | 0.0574 | 0.1054 | 0.0204 | 0.0276 | 0.0240 | 0.0072 |
| 10(C ) | 0.0286 | -0.0271 | 0.0578 | 0.0292 | 0.0557 | 0.0425 | 0.0265 |
| 11(C ) | 0.1202 | 0.0376 | 0.1295 | 0.0093 | 0.0826 | 0.0459 | 0.0732 |
| 12(C ) | -0.0666 | -0.0757 | -0.0446 | 0.0221 | 0.0091 | 0.0156 | -0.0129 |
| 13(C ) | 0.0540 | -0.0048 | 0.0671 | 0.0131 | 0.0588 | 0.0359 | 0.0457 |
| 14(C ) | 0.1916 | 0.1666 | 0.2054 | 0.0137 | 0.0250 | 0.0194 | 0.0113 |
| 15(O ) | -0.3045 | -0.3639 | -0.2703 | 0.0343 | 0.0594 | 0.0468 | 0.0251 |
| 16(O ) | -0.1916 | -0.2494 | -0.1649 | 0.0267 | 0.0578 | 0.0422 | 0.0311 |
| 17(O ) | -0.2594 | -0.3239 | -0.2323 | 0.0270 | 0.0645 | 0.0458 | 0.0375 |
| 18(N ) | -0.0718 | -0.0896 | 0.0605 | 0.1323 | 0.0178 | 0.0751 | -0.1145 |
| 19(C ) | -0.0097 | -0.0166 | 0.0120 | 0.0218 | 0.0069 | 0.0143 | -0.0148 |
| 20(C ) | -0.0147 | -0.0185 | -0.0041 | 0.0106 | 0.0037 | 0.0072 | -0.0069 |
| 21(N ) | -0.1063 | -0.1093 | -0.0989 | 0.0074 | 0.0030 | 0.0052 | -0.0044 |
| 22(C ) | -0.0143 | -0.0192 | -0.0007 | 0.0137 | 0.0049 | 0.0093 | -0.0088 |
| 23(C ) | -0.0115 | -0.0184 | 0.0131 | 0.0246 | 0.0069 | 0.0158 | -0.0176 |
| 24(C ) | -0.0439 | -0.0505 | -0.0313 | 0.0126 | 0.0066 | 0.0096 | -0.0060 |
| 25(C ) | -0.0852 | -0.0955 | -0.0786 | 0.0066 | 0.0103 | 0.0085 | 0.0038 |
| 26(F ) | -0.0901 | -0.1248 | -0.0590 | 0.0311 | 0.0347 | 0.0329 | 0.0036 |
| 27(H ) | 0.0550 | 0.0348 | 0.0718 | 0.0168 | 0.0202 | 0.0185 | 0.0034 |
| 28(H ) | 0.0431 | 0.0293 | 0.0601 | 0.0170 | 0.0137 | 0.0154 | -0.0033 |
| 29(H ) | 0.0530 | 0.0357 | 0.0688 | 0.0158 | 0.0174 | 0.0166 | 0.0016 |
| 30(H ) | 0.0600 | 0.0306 | 0.0819 | 0.0219 | 0.0293 | 0.0256 | 0.0074 |
| 31(H ) | 0.0639 | 0.0369 | 0.0764 | 0.0126 | 0.0269 | 0.0197 | 0.0144 |
| 32(H ) | 0.1260 | 0.0931 | 0.1431 | 0.0171 | 0.0329 | 0.0250 | 0.0157 |
| 33(H ) | 0.0311 | 0.0298 | 0.0482 | 0.0171 | 0.0013 | 0.0092 | -0.0158 |
| 34(H ) | 0.0266 | 0.0140 | 0.0642 | 0.0377 | 0.0126 | 0.0251 | -0.0251 |
| 35(H ) | 0.0163 | 0.0131 | 0.0249 | 0.0086 | 0.0032 | 0.0059 | -0.0053 |
| 36(H ) | 0.0363 | 0.0254 | 0.0575 | 0.0212 | 0.0109 | 0.0160 | -0.0104 |
| 37(H ) | 0.0375 | 0.0254 | 0.0610 | 0.0235 | 0.0121 | 0.0178 | -0.0114 |
| 38(H ) | 0.0167 | 0.0114 | 0.0291 | 0.0124 | 0.0053 | 0.0088 | -0.0071 |
| 39(H ) | 0.0249 | 0.0122 | 0.0642 | 0.0393 | 0.0127 | 0.0260 | -0.0265 |
| 40(H ) | 0.0372 | 0.0323 | 0.0536 | 0.0165 | 0.0049 | 0.0107 | -0.0116 |
| 41(H ) | 0.0357 | 0.0269 | 0.0502 | 0.0145 | 0.0087 | 0.0116 | -0.0058 |
| 42(H ) | 0.0351 | 0.0269 | 0.0491 | 0.0140 | 0.0082 | 0.0111 | -0.0058 |
| 43(H ) | 0.0178 | 0.0109 | 0.0304 | 0.0126 | 0.0069 | 0.0097 | -0.0057 |
| 44(H ) | 0.0372 | 0.0312 | 0.0368 | -0.0004 | 0.0060 | 0.0028 | 0.0065 |
| 45(H ) | 0.0431 | 0.0323 | 0.0557 | 0.0126 | 0.0108 | 0.0117 | -0.0018 |
| 46(H ) | 0.0436 | 0.0248 | 0.0560 | 0.0124 | 0.0188 | 0.0156 | 0.0064 |

Nucleophilic attack **:** f^+^= q_(N)_- q_(N+1)_ Electrophilic attack : f^-^= q_(N-1)_-q_(N)_  Radical attack: f^0^= (q_(N-1)_- q_(N+1)_)/2

**Table S4. The molecular interaction between AlgW with Andrographolide and Levofloxacin complex.**

| Compound ID | Glide GScore | Molecular interaction amino acids | Distance (Å) |
| --- | --- | --- | --- |
| Andrographolide | -8.030 | LEU282 | 3.2 |
|  |  | GLY283 | 3.2 |
|  |  | GLU285 | 5.6 |
| Levofloxacin | -3.261 | LEU282 | 3.1 |
|  |  | GLY283 | 3.0 |
|  |  | VAL284 | 2.8 |
|  |  | ARG374 | 3.1 |
